# Targeting the Zinc Transporter ZIP7 in the Treatment of Insulin Resistance and Type 2 Diabetes

**DOI:** 10.3390/nu11020408

**Published:** 2019-02-15

**Authors:** John Adulcikas, Sabrina Sonda, Shaghayegh Norouzi, Sukhwinder Singh Sohal, Stephen Myers

**Affiliations:** College of Health and Medicine, School of Health Sciences, University of Tasmania, TAS 7005, Australia; johna6@utas.edu.au (J.A.); sabrina.sonda@utas.edu.au (S.S.); shaghayeg.norouzi@utas.edu.au (S.N.); sukhwinder.sohal@utas.edu.au (S.S.S.)

**Keywords:** zinc, zinc transporters, ZIP7, insulin resistance, endoplasmic reticulum stress, type 2 diabetes

## Abstract

Type 2 diabetes mellitus (T2DM) is a disease associated with dysfunctional metabolic processes that lead to abnormally high levels of blood glucose. Preceding the development of T2DM is insulin resistance (IR), a disorder associated with suppressed or delayed responses to insulin. The effects of this response are predominately mediated through aberrant cell signalling processes and compromised glucose uptake into peripheral tissue including adipose, liver and skeletal muscle. Moreover, a major factor considered to be the cause of IR is endoplasmic reticulum (ER) stress. This subcellular organelle plays a pivotal role in protein folding and processes that increase ER stress, leads to maladaptive responses that result in cell death. Recently, zinc and the proteins that transport this metal ion have been implicated in the ER stress response. Specifically, the ER-specific zinc transporter ZIP7, coined the “gate-keeper” of zinc release from the ER into the cytosol, was shown to be essential for maintaining ER homeostasis in intestinal epithelium and myeloid leukaemia cells. Moreover, ZIP7 controls essential cell signalling pathways similar to insulin and activates glucose uptake in skeletal muscle. Accordingly, ZIP7 may be essential for the control of ER localized zinc and mechanisms that disrupt this process may lead to ER-stress and contribute to IR. Accordingly, understanding the mechanisms of ZIP7 action in the context of IR may provide opportunities to develop novel therapeutic options to target this transporter in the treatment of IR and subsequent T2DM.

## 1. Introduction

The prevalence of obesity has become pandemic due to an increasing obesogenic environment including poor nutrition and lack of exercise. The consequences of increased weight gain and obesity can result in the most devastating complications of type 2 diabetes mellitus (T2DM) [1]. The aetiology of T2DM is highly complex and multifaceted but in its simplest form, is caused by insulin resistance (IR), hyperglycaemia, pancreatic dysfunction and beta-cell failure [2].

IR is a systemic disorder that is broadly defined as the compromised ability of insulin to regulate glucose homeostasis in peripheral tissues. IR affects all cell types and is asymptomatic and therefore this makes it extremely difficult to treat and prevent [3]. Long-term IR leads to several chronic disease states including diabetes, cardiovascular disease and stroke. Moreover, treatments for IR have not advanced significantly in the last few decades because of the inadequate knowledge about the pleiotropic effects that these drugs have on specific molecular targets [2]. IR is typically present for several years before T2DM diagnosis and a foremost concern for individuals with IR is the progressive failure of pancreatic β-cell function and compromised insulin secretion. Therefore, prevention strategies that take advantage of this “window of opportunity” to prevent or lessen disease progression would have a significant health impact on people with this disorder. Accordingly, strategies targeting IR with novel molecules that increase the efficacy and safety of therapeutic treatment options are critical. 

A major factor in the pathogenesis of IR is endoplasmic reticulum (ER) stress. This is a condition where the ER becomes dysfunctional due to an imbalance of influx of nascent unfolded polypeptides exceeding the normal processing capacity of the ER [3]. This results in the activation of the unfolded protein response (UPR), a process that monitors the ER environment for compromised ER protein-folding capacity and relays this information to gene expression programs to ameliorate the ER stress response [4]. Prolonged ER-stress leads to constitutive UPR activity and compromised mitigation of ER homeostasis leading to apoptosis and cell death. 

While research to elucidate mechanisms implicated in ER-stress and IR are ongoing, recent studies have identified a role for zinc and the protein transporters of this metal ion in ER stress and apoptosis [5]. Zinc is an essential metal ion that is critical for maintaining cellular function and homeostasis [6] and dysregulation of zinc-mediated action causes many abnormalities and disease states in humans and animal models [7]. Zinc is transported into and out of cells by an elaborate family of zinc transporter proteins that control subcellular organelle zinc and cytosolic intracellular zinc concentrations [8]. Recently, zinc and the proteins that transport this metal ion have been linked to ER stress and subsequent disease states including IR and T2DM [3,5,9,10,11,12,13]. Zinc is stored in the ER and evidence suggests that this metal ion is required for homeostatic functions of this organelle [9]. One zinc transporter that has gained attention in ER-stress and IR-related research is ZIP7 [12,13,14,15,16]. This transporter has been coined the “gate-keeper” of zinc release from the ER into the cytosol and initiates the subsequent zinc-mediated activation of cell signalling pathways that promote cell proliferation and cellular homeostasis [17]. Accordingly, it is tempting to speculate that ZIP7 may be involved in ER homeostasis and mechanisms that disrupt the ER and ZIP7 function may lead to IR. Thus, strategies to target ZIP7 therapeutically may be beneficial in the treatment of IR and thus T2DM.

## 2. Insulin Resistance and Type 2 Diabetes 

T2DM is a devastating disease characterized by IR and pancreatic beta cell failure [2]. Preceding the development of T2DM is a series of disordered metabolic states including elevated fasting insulin, obesity, dyslipidaemia and IR [18,19]. IR is usually characterized by compromised insulin sensitivity and dysregulated glucose homeostasis in peripheral tissues including adipose, liver and skeletal muscle [20,21]. Over the course of the disease, T2DM and its complications (for example renal dysfunction and retinal damage) [21] continuously worsen and initial drug therapy is replaced by polypharmacy [21,22]. Therefore, identifying opportunities to develop novel therapeutic strategies to ameliorate IR before the development of T2DM is critical. 

Insulin acts on multiple cellular processes that essentially provide an integrated molecular response to maintain glucose homeostasis between nutrient supply and demand [23]. In normal physiology, this response is maintained through the mobilization of glucose transporters to the plasma membrane by stimulating the canonical IRS-PI3K-AKT pathway [19]. The simplified mechanism of insulin action proceeds through the activation of the insulin receptor upon binding insulin. The insulin receptor consists of two extracellular α subunits and two transmembrane/intracellular β subunits [24]. A central feature following the binding of insulin to the insulin receptor is the activation of the intrinsic tyrosine kinase that initiates autophosphorylation of the receptor [24]. This leads to the phosphorylation of insulin receptor substrates (IRS-1 and IRS-2) which promotes the activation of PI3K and protein kinase B (PKB)/AKT [25]. The post-prandial actions of insulin include the mobilization of glucose transporters to the plasma membrane in muscle, cardiomyocytes and adipose tissue, activation of glycogen synthase and thus glycogen synthesis and inhibition of gluconeogenesis in hepatocytes and inhibition of lipolysis in adipose tissue [25,26]. 

In IR, target cells that have a high metabolic demand for glucose (liver, adipose tissue and skeletal muscle) [27] fail to respond to physiological levels of circulating insulin and thus, higher concentrations of insulin are required to exert a response [23,28]. While IR is highly complex and the precise pathophysiology is not clear, several molecular mechanisms are known to be implicated in this disorder. These include but are not limited to, defects in the phosphorylation of the insulin receptor and downstream phosphorylation of AKT, reduced capacity of insulin to bind to the insulin receptor, mutations in glucose transporter proteins, increase in inflammatory mediators (e.g. TNF-α and interleukins), free radical overload, mitochondrial dysfunction and ER stress [28] (and references herein). 

### Endoplasmic Reticulum Stress and Insulin Resistance

It is well established that ER stress is a major contributor to many disease states including obesity, IR and T2DM [29,30] (and references therein). The ER performs vital functions in the synthesis, folding and transport of proteins and represents a major manufacturing and processing “node” in cellular function. Dysregulation of this “node” through ER stress, for example, compromises the cellular integrity of the ER and protein folding processes. While the ER has mechanisms to compensate for misfolding protein events and attempts to re-establish the homeostatic control of the ER folding capacity, increased misfolded protein load exceeds the ability of the ER to maintain control and thus, ER stress is initiated [31]. To alleviate ER stress and correct protein folding activities, the ER activates the unfolded protein response (UPR). Thus, the ER adapts to misfolding events by reducing the rate of protein synthesis and activates transcriptional regulation of UPR genes that are implicated in ER protein folding [30,31]. Finally, if ER homeostasis cannot be restored, cell death is activated. 

While the processes of ER stress in the development of IR has been discussed in previous reviews, here we focus on a newly described role for zinc and the proteins that transport this metal ion in ER stress-related IR. For example, recent work has revealed that zinc depletion in yeast cells activated the ER-stress gene Ire1 [10]. Moreover, these studies demonstrated that zinc depletion activated the ER-localized chaperone BIP and suggested that protein folding in the ER was impaired. More recently, the zinc transporter ZIP14 was identified as playing an important role in suppressing ER stress-induced apoptosis and hepatic steatosis [5]. Similarly, the zinc transporter ZIP7 was suggested to maintain zinc homeostasis in the ER in human MG-63 osteosarcoma cells [11], human KBM7 myeloid leukaemia cells [32] and intestinal epithelial cells [13]. Accordingly, interventions targeting ZIP7 might be amenable to clinical intervention to ameliorate ER stress and modify existing complications associated with IR.

## 3. Zinc Transporter Proteins 

Zinc is unable to pass effectively through the plasma membrane and therefore requires several protein transporters to facilitate its flux. Zinc transporter proteins are responsible for maintaining organelle and cytosolic zinc concentrations and exist in two distinct families: solute carrier family 30A (SLC30A) and 39A (SLC39A) [33] that play a critical role in zinc uptake and transport across biological membranes [8].

### 3.1. SLC30A Family 

**Figure 1:**
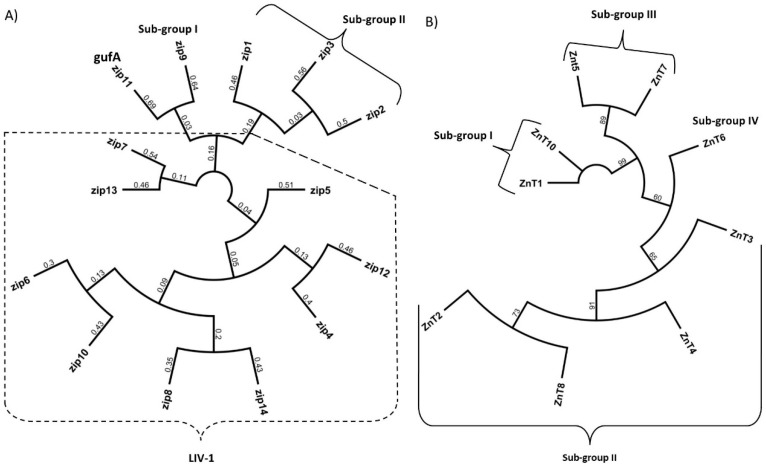
Phylogenetic relationships of the human ZnT and ZIP proteins (SLC30A & SLC39A family of zinc transporters). Protein sequences were attained by converting human SLC39A and SLC30A mRNA into amino acids sequences and aligned by Geneious alignment using global alignment. The dendrogram was generated from Geneious tree builder utilizing the Jack-knife method. Molecular phylogenetic analysis as generated by the genetic distance model “Jukes-Cantor”, the re-sampling method used Jack-knife with 229,871 random seeds. (A) Phylogenetic relationships of SLC39A family (ZIP), (B) Phylogenetic relationships of SLC30A family (ZnT).

The SLC30A family of zinc transporters are part of the vertebrate cation diffusion facilitator proteins that are made up of three subgroups based on the cation they transport, namely Zn, Zn/Fe and Mn. In mammals, ten SLC30A are described (named ZnT1–ZnT10) [34] all of which have a predicted six transmembrane domains (TMD) with a histidine/serine loop between TMDIV and TMDV [35] that could function as a metal binding domain [36]. ZnT9 remains to be shown to function as a zinc transporter [37] resulting in nine confirmed SLC30A members that transport zinc out of the cytoplasm either into subcellular organelles and vesicles or extracellularly. The SCL30A family is further divided into four sub-groups based on their sequence similarities as follows: Subgroup I: ZnT1 and ZnT10, Subgroup II: ZnT 2, 3, 4 and 8, Subgroup III: ZnT5 and ZnT7 and Subgroup IV: ZnT6 [38] (Figure 1).

### 3.2. SlC39A Family 

The solute carrier family 39A (SLC39A) of zinc transporters, increase cytosolic zinc by transport of this metal ion into the cell via the plasma membrane or from subcellular organelles [14]. In mammals, fourteen members of this family have been described (named ZIP1-ZIP14) [39] all of which have a predicted eight TMDs and many members of this family also have a long histidine-rich loop region between TMDIII and TMDIV that potentially binds zinc [40]. The SLC39A family is separated into four sub-groups based on amino acid sequence similarities [16]; Group I: ZIP9, Group II: ZIP1, 2 and 3, gufA: ZIP11 and LIV-1: ZIP 4–8,10 and 12–14 (Figure 1). In addition to transporting zinc, SLC39A members were shown to transport a wide range of cations. For example, ZIP14 and ZIP8 have a high affinity for Cd^2+^, Mn^2+^ and Fe^2+^ and are involved in the transport of these ions across membranes [41,42].

The importance of the zinc transporter family is highlighted by the numerous functions of these proteins in many disease states [39,40,43,44,45,46]. Dysregulation of these transporters results in decompartmentalization and fluctuations in intracellular zinc which affect multiple signalling pathways involved in normal development, growth, differentiation and death [46] and have profound effects on metabolic homeostasis leading to pathophysiological effects [8,47]. 

## 4. Zinc 

Zinc is a vital trace element that is crucial for its role in supporting normal physiological function and cellular homoeostasis. As a divalent cation, zinc has numerous roles in catalytic [48], cell regulatory/signalling pathways [49,50] and structural components of many proteins [51] that underpin various physiological and cellular functions including those of the immune, reproductive, endocrine, skeletal and neuronal systems. Zinc’s regulatory role is a dynamic process where an increase in cytosolic labile zinc concentration facilitates the activation of a wide range of cell signalling pathways including MAPKs, such as ERK1/2, JNK and p38, tyrosine kinases Src, EGFR [52], insulin receptor substrates 1 and 2 (IRS1/2) and IGF1 receptor [53,54]. Zinc also provides structural integrity to many proteins [51] and is a cofactor for more than three-hundred enzymes [48]. 

Intracellular “free” zinc is sequestered within organelles such as the ER, Golgi and mitochondria with little “free” zinc found within the cytoplasmic [55], thereby, these organelles serve as reservoirs for zinc storage. In this context “free” indicates unbound zinc (in contrast to protein-bound zinc) which depends on the zinc buffering capacity of the cell [15]. While only small concentrations of “free” zinc exist in a cell, it is unquestionably not insignificant. In fact, it is vital for the control of many cell signalling processes and parallels the actions of calcium signalling [56]. 

Within organelles and vesicles, the concentration of zinc is thought to range between tens and hundreds of micromolar [36,57,58]. Unlike iron or copper that require redox reactions to cross cellular membranes, zinc does not, resulting in its use without the risk of oxidative damage [59]. Zinc’s lack of redox reaction also makes it a suitable structural component within gene regulatory proteins as other redox active ions can generate free radicals that may subsequently damage DNA and RNA (references herein [60]). Also, zinc is reactive as a Lewis acid and together with zinc’s flexible coordination geometry, this makes zinc ubiquitous in subcellular metabolism and invaluable to biological systems [61]. Hence over 2800 proteins [48] of which, 300 are enzymes are thought to bind zinc thereby making zinc the second most abundant essential metal after iron [62].

## 5. Pathophysiology and Pathogenesis of Dysfunctional Zinc Homeostasis 

The dysfunctional regulation of zinc homeostasis and associated pathophysiology has been studied extensively. While these have been covered succinctly in other reviews [39,40,46,63] a brief overview of some of the more well-studied disease states merits some attention. 

Zinc plays a major role in embryonic, foetal and postnatal development in higher eukaryotes [34]. Zinc-deficiency symptoms are varied, affecting many body systems [37] (and references herein). Disturbances in zinc homeostasis are associated with several disease states including liver cirrhosis [64], growth retardation and acrodermatitis [65], tumours [66,67], immunity [68], bowel disease [69], Alzheimer’s disease [70] and diabetes [6,71]. Moreover, disturbances in zinc homoeostasis have been linked to the dysfunction or dysregulation of zinc transporter proteins. Suffice to say, zinc transporter proteins play a pivotal role in zinc homoeostasis. For example, prostate cancer patients had reduced mRNA and protein expression of ZIP1 and ZIP3 (SLC39A transporters) and reduced intracellular zinc [72]. Moreover, these studies showed that overexpression of ZIP1 in prostate cancer cell lines increased intracellular zinc and limited cancer cell growth due to increased apoptosis. 

A range of neurodegenerative conditions has also been observed in patients with altered expression of zinc transporters [73,74]. For example, ZnT3 and ZnT10 transporter levels are significantly reduced in Alzheimer’s disease brain and suggest that dysfunctional zinc handling may be an early event in the pathogenesis of this disease [74]. Mutations in the ZnT2 transporter gene can cause transient neonatal zinc deficiency that is characterised by low zinc levels in breast milk and only exhibits while weaning. Dietary zinc supplements taken by the mother does not increase milk zinc levels whereas zinc given directly to the infant alleviates zinc deficiencies [75,76]. Spondylocheirodysplastic Ehlers-Danlos syndrome (SCD-EDS), a condition that displays postnatal growth retardation, bruised and hyperelastic skin and tissue abnormalities are attributed to homozygous loss-of-function mutations of ZIP4 and ZIP13, respectively [77]. 

### Zinc Transporters and Diabetes

Of the zinc transporters, perhaps the most well-studied is ZnT8. This transporter is predominately expressed in the endocrine pancreas and specifically enriched in the β-cells [78]. ZnT8 co-localizes with insulin and is consistent with its role in facilitating zinc uptake into the insulin secretory granules [78]. This process is critical for the maturation and crystallization of insulin where insulin is stored as hexamers with zinc before secretion [36]. Due to the tissue-specific expression of this transporter, ZnT8-null mice display compromised β-cell function, reduced insulin secretion and low circulating insulin levels and impaired glucose tolerance [79,80,81,82]. In humans, polymorphisms in ZnT8 are associated with type 1 and type 2 diabetes mellitus. For example, human genome-wide association studies on 392,935 genotypes identified a single nucleotide polymorphism (SNP) corresponding to rs13266634 (a C/T polymorphism) which encodes either arginine (R) by the C allele or tryptophan (W) by the T allele and results in a missense mutation substitution R325W that confers T2DM risk [83,84]. Testing of this SNP in transgenic mice overexpressing the human ZnT8 wild-type or the human ZnT8 R325W identified reduced pancreatic zinc concentrations and decreased insulin and glucose tolerance in the mutant mouse model [85]. 

Similarly, a Diabetes Prevention Program study that enlisted 3234 USA participants at high risk of developing T2DM were screened for the ZnT8 rs13266634 SNP. Of the 3007 participants that were genotyped, it was found that the C risk allele was significantly associated with elevated proinsulin levels [86]. Despite having the C risk allele, those patients that were placed on treatment (metformin or a lifestyle intervention) for one year were no longer associated with proinsulin levels and suggest that the ZnT rs13266634 SNP activity could be ablated by preventative treatment prior to the development of T2DM [87]. 

Recently, twelve rare ZNT8 truncated (loss-of-function) variant proteins were identified in a large population across five ethnicities that conferred a 65% reduced risk of T2DM [88]. Of the rare mutations, two most common protein-truncating variants (p.Arg138X and p.Lys34SerfsX50) encode unstable ZnT8 proteins and individually associate with T2DM protection [88]. While studies in mice have identified that the R325 variant display lower transport activity than the W325 [85] and reduced zinc transport increases T2DM risk [89], the loss of function ZnT8 mutations in humans provides strong evidence that ZnT8 haploinsufficiency protects against T2DM [88].

ZnT8 is also a major autoantigen in the development of autoimmunity in type 1 diabetes (T1DM) [35]. Autoantibodies to ZnT8 (ZnT8A) are detected in 60% to 80% of early-onset T1DM and approximately 30% of patients with other autoimmune disorders [90]. A previous report delineated key epitopes implicated in T1DM autoimmune response to ZnT8 [91]. This study found that the amino acid encoded by the common polymorphism in human ZnT8 (rs13266634; R325) is a key determinant of two of the three major conformational epitopes in the transporter. The authors suggest that while the rs13266634 genotype or epitope specificity of ZnT8 may not contribute to T1DM susceptibility or age of onset, the clinical implications will be important for antigen-based therapeutic interventions. Similarly, studies in Japanese patients with T1DM identified ZnT8 autoantibodies in 58% of patients with acute-onset and in 20% with slow-onset T1DM [92]. However, none of the sera was reactive to ZnT8 from fulminant T1DM patients and suggests that ZnT8 autoantibodies are useful as a diagnostic marker for acute onset but not fulminant diabetes [92]. 

The remainder of this review will address the importance of zinc, ZIP7, ER stress and insulin resistance. Where possible, extrapolation from other zinc transporters will be discussed where there is a direct link between ER stress and/or IR.

## 6. ER Stress, Zinc and Insulin Resistance 

The endoplasmic reticulum is of interest as it acts as a mobilizable zinc store with concentrations of this metal exceeding 5 nM [93]. The ER is critical for the correct processing and folding of proteins and ER stress arises when the folding capacity of the ER is outpaced with the influx of nascent, unfolded polypeptide chains. Perturbations that lead to ER stress can occur under normal biological conditions such as the differentiation of B cells into plasma cells within *C. elegans* [94] and from the folding of heavily modified proteins that are overexpressed, as with blood coagulation factor VIII [95]. Alternatively, expression of mutant genes, ischemia, hypoxia, oxidative stress or uncharacteristic increased in protein synthesis can lead to ER stress and the activation of the UPR which is the basis of several diseases including diabetes, haemophilia [96], neurodegenerative disease [97,98] and cystic fibrosis [99]. 

The overarching function of the UPR is to relay the protein-folding status in the ER [100] (and references herein). The UPR’s role goes beyond surveillance and has been linked to nutritional [101], differentiation [102] or infection status of the cell [98]. Cells undergoing ER stress will activate the UPR pathway that downregulates transcription of genes that code for secretory proteins, to lower the folding demand on the ER [103]. Along with taking measures to initiate the UPR to reduce ER stress, the cell can also increase the size and volume of the ER to dilute misfolded proteins [104]. 

Recently, zinc and the transporters of this metal ion were shown to be involved in the development of ER stress [5,10,12,13] and have also been linked to insulin resistance and type 2 diabetes [6,8,47,105]. For example, a lack of zinc when used as a cofactor can adversely affect the metalation of certain ER-resident proteins/enzymes thereby affecting their correct function [106,107], thus inducing ER stress. Zinc as an ion will not exist in a free state within biological systems and will always be in coordination with a charged/polar residues such as aspartic acid, glutamic acid, cysteine and histidine, all of which have a high affinity for zinc [59]. Therefore, zinc binding these sites may cause misfolded, non-functional or semi-functional proteins [106]. For example, the DNA-binding domain of the tumour suppressor p53 can undergo misfolding in the presence of low concentrations of zinc [108]. However, little is known about ER zinc storage and cellular zinc homoeostasis, only that the ER, like many sub-organelles, is a zinc sequestration site for cytoplasmic zinc [109].

Within the cytoplasm, zinc is managed by the transport and subsequent sequestering of zinc from the cytosol into subcellular stores or labile cytoplasmic zinc is bound up with special metal ion binding proteins called metallothioneins [110]. Accordingly, steady states of “free” intracellular zinc ions are buffered within narrow limits and are under tight regulatory control due to the enormous number of proteins and enzymes that require zinc to function [111,112]. The metallothioneins preferentially bind zinc over other metals and therefore binding of zinc to these proteins cannot occur in a promiscuous manner as this would render many metalloproteins dysfunctional [112]. The cell has evolved to circumvent this problem through the compartmentalization of intracellular zinc. Thus, the modulation of subcellular organelle zinc and cytosolic zinc concentrations are achieved by the elaborate zinc transporter family of proteins and metalloproteins in what has been termed “muffling” reactions [111,112]. 

Accordingly, dysfunctional regulation of the zinc transporter proteins and the decompartmentalization of intracellular zinc, through initial ER stress events or other factors that involve zinc transporter-mediated ER stress might exacerbate this condition leading to the UPR and apoptosis. For example, recent studies in mice suggest that dietary zinc is essential of ER stress-induced apoptosis [113]. Mice challenged with tunicamycin and fed dietary zinc (1 mg zinc/kg) had increased expression of ER stress-related markers p-eIF2α, ATF4 and CHOP while those mice fed 180 mg zinc/kg had less expression of these markers [113]. These results suggest that zinc-mediated ER stress adaptation is initiated via the repression of the proapoptotic pathway of the UPR. 

## 7. The Zinc Transporter ZIP7 in Endoplasmic Reticulum Stress and Insulin Resistance 

ZIP7 has been extensively studied in a number of cell-based systems and animal studies. It is most documented for its role in cancer studies. For example, ZIP7 is upregulated in human cervical and colorectal cancer cells and silencing of ZIP7 suppressed cell proliferation, migration and invasion of these cells and induced apoptosis [114,115]. ZIP7 has also been associated with neuronal ceroid lipofuscinoses, a group of fatal childhood neurodegenerative lysosomal storage diseases that have no cure [116]. These studies demonstrated an age-related decline in CLN6 (an ER resident protein involved in protein processing and transport) in the brains of Merino sheep that was also associated with a loss of ZIP7. It was suggested that aberrant decompartmentalization of zinc in CLN6 disease is associated with a direct loss of ZIP7. While other studies on the role of ZIP7 (and the other zinc transporters) in disease processes are emerging and have been extensively reviewed elsewhere [37,39] (and references therein), the role of ZIP7 in insulin resistance and type 2 diabetes is novel and has not been widely studied. 

ZIP7 like all SLC39A members, share many conserved regions predominantly within the TMDs and histidine-rich motifs found within the LIV-1 sub-group. The N-terminal region of many LIV-1 members harbours several histidine residues however, ZIP7 has a statistically significantly high proportion of histidine within the N-terminal region relative to the SLC39A family [15]. The function of this high number of histidine residues has not been determined however, the tendency for histidine to be used to bind zinc hints at a yet undiscovered relationship between ER stored zinc and ZIP7.

ZIP7 is unique in that it is localized to the early secretory pathway including the ER and Golgi apparatus and functions as a zinc “gatekeeper” that tightly controls the movement of zinc from these subcellular organelles into the cytosol in what is termed the zinc “wave” the [7]. The phosphorylation of ZIP7 at Ser275 and Ser276 by casein kinase 2 (CK2) is thought to activate the gated release of zinc into the cytosol from the ER in human MCF-7 breast cancer cells [17]. These studies demonstrated that ZIP7 and CK2 co-precipitated in zinc-treated MCF-7 cells and suggest a zinc-dependent association between these two proteins. Moreover, inhibition of CK2 with dimethylamino-4,5,6,7-tetrabromo-1H-benzimidazole (DMAT) or a CK2-specific siRNA decreased the phosphorylation of ZIP7. This was concomitant with a reduction in zinc flux in cells treated with DMAT [17] and suggests an association between CK2 and ZIP7 phosphorylation is required for the gated released of zinc into the cytosol. The zinc wave is fundamentally different from standard zinc homeostatic processes taking place elsewhere in the cell. In that, the zinc wave is triggered in response to the phosphorylation of ZIP7 with the purpose to mediate cell signalling processes [16,17] as opposed to maintaining zinc levels. Accordingly, the importance of ZIP7 is emerging as a key transporter implicated in zinc efflux and maintenance of ER homeostasis.

ZIP7 was shown to be critical for maintaining ER homeostasis in MG-63 osteosarcoma cells [11]. These studies found that loss of ZIP7 caused decreased cell proliferation and ER stress as evidenced by a significant increase in the protein disulphide isomerase-A1 (PDI), a protein involved in oxidative folding [117]. Moreover, consistent with ER stress, the ZIP7 null cells exhibited strong expression of C/EBP homologous protein, CHOP [11]. This transcription factor, localized to the cytosol, is mobilized and translocated to the nucleus during ER stress and regulates stress-mediated cell death [118]. Furthermore, loss of ZIP7 in MG-63 cells resulted in decreased cytosolic zinc and a shift of the equilibrium toward increased ER zinc status, while overexpression of ZIP7 ameliorated ER stress with a significant reduction in CHOP and PDI [11]. 

These results for ZIP7 are further support by studies in intestinal epithelial cells where zinc transporter is implicated in ER homeostasis [13]. These studies identified that ZIP7 deficiency in transit-amplifying cells, which are localized to the lower part of the crypt of the intestine, induced ER stress and apoptosis [13]. These data suggest that ER-localized zinc is fundamentally regulated by ZIP7 and factors that induce ER stress might compromise ZIP function, exacerbating ER stress and apoptosis. Other studies to support a role for ZIP7 in ER stress identified that hyperglycemia-induced changes in embryonic rat heart-derived cells (H9c2) result in a redistribution of cellular zinc in the ER that involved associated changes in ZIP7 levels and ZIP7 phosphorylation [12]. 

While several recent studies have emerged linking ZIP7 to ER homeostasis and ER stress, other zinc transporters are now sharing some attention. For example, ZIP14 was shown to be critical for zinc uptake in liver and adaptation to ER stress [5]. In this study, tunicamycin, a potent ER stress inducer, regulated the increased expression of ZIP14 in mouse liver. Moreover, these mice displayed compromised zinc uptake in the liver and increased apoptosis during tunicamycin-induced stress. These studies also showed a significant upregulation of ZIP14 by ATF4 and ATF6α (ER-stress markers) and suggest that these transcription factors increase the expression of ZIP14 leading to hepatic zinc uptake and suppression of ER-stress induced apoptosis. 

## 8. ZIP7 and Glucose Homeostasis

**Figure 2:**
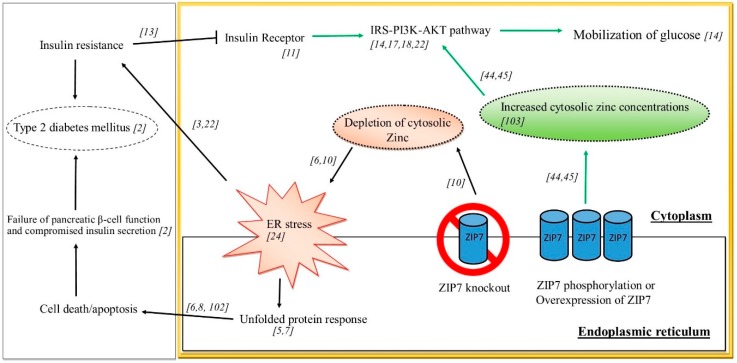
Schematic diagram of ZIP7 and its role in regulating pathways involved in glucose mobilization, ER stress, IR and T2DM.

Understanding the role of ZIP7 in glucose homeostasis and subsequent IR and T2DM is currently limited by existing studies and a lack of suitable animal models, although some extrapolation from cell-based studies and other related disease states may help elucidate a role for this zinc transporter in controlling glucose homeostasis. Previously it was identified that ZIP7 mRNA was elevated in response to glucose in mouse pancreatic islets [119]. Similarly, ZIP7 phosphorylation were increased in cardiomyocytes from diabetic rats and in H9c2 rat cardiomyocyte cells treated with high glucose concentrations mimicking chronic glycemia [12]. It was further demonstrated by these authors that the redistribution of cellular free zinc in hyperglycaemic cardiomyocytes was dependent on the phosphorylation of ZIP7. Other studies utilizing siRNA, demonstrated that reduced ZIP7 in mouse C2C12 skeletal muscle cells led to a reduction in the expression of a number of glucose metabolic genes and proteins including the insulin receptor, the phosphorylation of AKT and decreased Glut 4 protein expression and this was concomitant with a decrease in glycogen synthesis [14]. These above studies suggest a role for ZIP7 in modulating metabolic processes associated with controlling glucose metabolism. Highlighting the role of cellular events associated with zinc signalling, recent studies demonstrated that zinc activated several proteins (PRSA40, AKT, ERK1/2, SHP-2, GSK-3β and p38) in both mouse and human skeletal muscle cells that was concomitant with an increase in glucose oxidation [120]. It appears that zinc and ZIP7 are emerging as important factors that have roles in ER stress and IR. The studies elucidating a role for ZIP7 in these processes is depicted in Figure 2. Thus, phosphorylation and/or overexpression of ZIP7 in the ER leads to increased cytosolic zinc concentrations that activated cell signalling pathways (e.g. IRS-PI3K-AKT), stimulating glucose mobilisation and uptake. Similarly, overexpression of ZIP7 inhibits ER stress (not shown in the scheme). Studies on ZIP7 knockout have identified depletion of cytosolic zinc and the concomitant activation of ER stress which can cause IR and T2DM.

## 9. Conclusions 

Insulin resistance is a condition in which an organism fails to respond to endogenous insulin. Consequently, glucose uptake is compromised in tissues such adipose and skeletal muscle. IR is associated with the development of type 2 diabetes mellitus which accompanies several pathological conditions including obesity, cancer and neurodegenerative disease. Despite years of research, there is still great uncertainty regarding the molecular mechanisms of IR. Human gene mutations in insulin signalling pathways trigger IR, however most IR people do not harbour these mutations, so attention has focused on other causes of IR, including endoplasmic reticulum stress.

The ER plays a significant role in protein homeostasis through its functions related to the synthesis, folding and transport of proteins. Data supporting the contribution of defective insulin signalling in the development of ER stress-linked IR is strong, however the molecular mechanisms by which this effect occurs is lacking. ER stress triggers a signalling cascade known as the UPR, an acute mechanism that aims to re-establish cellular homeostasis through adaptive programmes that improve protein-folding processes. Recently, a supporting role for the zinc transporter ZIP7 has suggested that this protein ameliorates ER-stress in heart muscle cells through ER-mediated zinc flux and therefore has a protective role against oxidative stress and IR. The spatiotemporal regulation of ER-mediated zinc flux is tightly maintained by ZIP7 and studies that delineate the molecular mechanisms whereby ZIP7 protects the ER from oxidative stress and subsequently ameliorates IR will have enormous therapeutic utility for the development of novel drugs targeting this transporter.
